# 

**Published:** 1902-11

**Authors:** 


### MARRIAGE.

At Pittsburg, Pa., Wednesday, October 29, 1902, Miss Clara F. Aull and Dr. J. Stewart Lacock. An uncle of the bride, Rev. Charles B. Mitchell, of Cleveland, Ohio, performed the marriage ceremony. Dinner was served and a large reception given. Dr. Lacock is a graduate of the Veterinary Department, University of Pennsylvania, and is located at Allegheny, Pa., at which place the couple will reside after returning from the wedding-trip of several weeks.

### PERSONALS.

Dr. Leonard Pearson, State Veterinarian, was the judge of cattle, and Dr. C. J. Marshall, of Philadelphia, the judge of horses, at the Oxford (Chester County) Fair in September. The single judging system was inaugurated at the Fair this year.

The *Gleaner*, a journal published by the National Farm School, reproduces in the October number an excellent photogravure of Dr. W. G. Benner, of Doylestown, Pa., one of the corps of instructors at that institution.

Dr. J. C. McNeil and his assistant, Dr. H. H. Myers, of Pittsburg, Pa., were on the rack in October before the Committee on Revisions of Salaries of the various departments of the city of Pittsburg, Pa.

Dr. John T. Claris, of Buffalo, N. Y., was the defeated candidate for sheriff on the Republican ticket of Erie County in the recent election. Dr. Claris was an active Democrat in the Cleveland administrations.

Dr. A. T. Sellers, of Camden, N. J., has added a boarding stable with hospital facilities in connection with his practice.

### BINDING OF THE JOURNAL.

THOSE contemplating binding their JOURNALS for the year 1902 in the half-sheep form adopted by the JOURNAL may now arrange for the same by writing to the office of this publication. We will bind in this form any number of volumes, and will endeavor to supply missing numbers at current rates wherever the same is possible. All orders must reach us not later than January 20, 1903. Send JOURNALS by express, prepaid.

